# Metastatic Carcinoma Occurring in a Gastric Hyperplastic Polyp Mimicking Primary Gastric Cancer: The First Reported Case

**DOI:** 10.1155/2014/781318

**Published:** 2014-10-22

**Authors:** Gabriel M. Groisman, Roman Depsames, Baruch Ovadia, Alona Meir

**Affiliations:** ^1^Institute of Pathology, Hillel Yaffe Medical Center, 38100 Hadera, Israel; ^2^Institute of Gastroenterology, Hillel Yaffe Medical Center, 38100 Hadera, Israel

## Abstract

Hyperplastic polyps of the stomach are regarded as benign. However, in rare cases they may contain incipient primary carcinomas. To our knowledge, breast carcinoma metastatic to a gastric hyperplastic polyp has not yet been reported. We describe the case of a 69-year-old woman to whom a gastric polyp was endoscopically excised. The patient had previously undergone a right mastectomy for mixed, invasive ductal and lobular carcinoma 5 years earlier. Histological sections from the gastric lesion showed typical features of hyperplastic polyp with foci of poorly differentiated adenocarcinoma including signet ring cells infiltrating the lamina propria. The histologic findings were consistent with a primary gastric cancer. However, the carcinoma cells were immunopositive for estrogen and progesterone receptors and GATA3 and negative for CDX2, Hep Par 1, and MUC5AC. E-cadherin showed membranous reactivity in some of the carcinoma cells while in others it was negative. Accordingly, metastatic mixed, lobular and ductal breast carcinoma was diagnosed. We conclude that metastatic adenocarcinoma mimicking primary gastric cancer can be rarely encountered in hyperplastic gastric polyps.

## 1. Introduction

Metastatic disease involving the stomach is a rare occurrence. In a series of 771 patients with gastric tumors found at endoscopy, only 2.6% were secondary neoplasms [1]. Although all primary malignancies can metastasize to the stomach, gastric metastases most often originate from malignant melanomas or carcinomas of the breast, lung, and esophagus [2]. Interestingly, two-thirds of metastatic mammary cancers to the stomach are of lobular type [3]. The most common clinical presentations of gastric metastases include anemia, gastrointestinal bleeding, abdominal pain, and dyspepsia [1]. The most common endoscopic appearance is that of a submucosal nodule seen as a mass with a smooth surface. Alternatively, metastatic lobular breast carcinoma may resemble advanced gastric cancer with features of linitis plastica [3, 4].

Hyperplastic polyps are common gastric lesions. Although they are regarded as nonneoplastic, development of primary adenocarcinoma may rarely occur within these polyps [5–8]. To our knowledge, metastatic adenocarcinoma to a gastric hyperplastic polyp has not been yet reported. In this report, we present the case of a hyperplastic gastric polyp containing metastatic breast carcinoma that simulated primary gastric cancer.

## 2. Case Presentation

**Figure 1:**
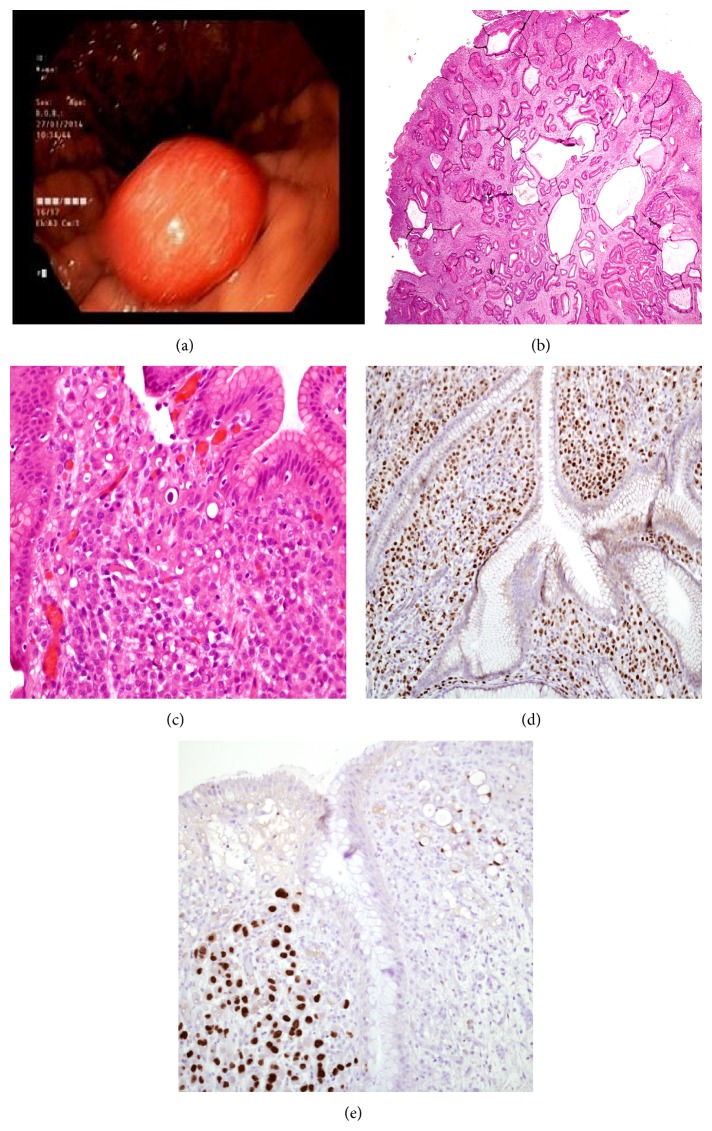
(a) Endoscopic view of gastric polyp. (b) Low power microscopic view of the excised lesion showing typical features of gastric hyperplastic polyp, namely, elongated, tortuous, and cystic foveolae separated by edematous and inflamed stroma (hematoxylin and eosin stained section, magnification ×20). (c) High power view shows carcinoma cells, including signet ring cells, infiltrating the lamina propria among benign gastric foveolae (hematoxylin and eosin stained section, magnification ×400). (d) Estrogen receptors and (e) GATA3 strongly stain the nuclei of the cancer cells while the gastric epithelial cells remain negative for both markers ((d) and (e) magnification ×200).

A 69-year-old woman with a five-year history of mixed ductal and lobular breast cancer was found to have a polypoid gastric mass on a CT scan and was sent to a gastroscopy. Five years previously, she underwent a right mastectomy for a mixed ductal (grade 2) and lobular invasive carcinoma. The tumor measured 4.5 cm in maximum diameter. Axillary dissection demonstrated that 15 of 17 lymph nodes contained metastases. The tumor was moderately positive (2+ of 3) for estrogen receptor and progesterone receptor (80% and 10% of cells, resp.) and negative (FISH technique) for Her 2-neu. As no other metastatic foci were found the stage was summarized as pT2N3M0. She was treated with chemoradiotherapy and hormonal therapy. After three years, a gastroscopy was performed for epigastric discomfort. No polyps were detected and antral biopsies showed chronic erosive gastritis with reactive changes and* Helicobacter pylori*. Eight months prior to the last endoscopy, she developed ascites. Cytological examination demonstrated the presence of carcinoma cells compatible with a breast origin. She was oncologically treated and a follow-up CT scan revealed resolution of the ascites and the presence of a gastric polyp. No evidence of metastatic disease was found. On gastroscopy several polypoid formations were detected. The largest one, measuring 2.0 cm in diameter, was excised (Figure 1(a)). The other polyps and the nonpolypoid mucosa were not biopsied. Six months following the procedure the patient is alive with evidence of widespread metastatic disease including recurrence of malignant ascites.

## 3. Pathological Examination

Gross examination of the endoscopically resected tumor revealed a round, smooth, red, soft mucosal polyp measuring 2.0 cm in diameter with a short stalk measuring 0.2 cm in height and 0.4 cm in diameter. Sections were embedded in paraffin and stained with hematoxylin and eosin. Immunohistochemistry using the streptavidin-biotin peroxidase complex method was performed on a Ventana Benchmark automatic immunostainer (Tucson, AZ, USA) with the following antibodies: cytokeratin 7 (clone OV-TL 12/30, ready to use [RTU]; Dako, Glostrup, Denmark), cytokeratin 20 (clone Ks 20.8, RTU; Dako), Hep Par 1 (clone OCH1E5, 1 : 25; Dako), GATA3 (clone 634913, 1 : 50; R&D Systems, Minneapolis, MN, USA), estrogen receptor (clone SP1, RTU; Ventana, Tucson, AZ, USA), progesterone receptor (clone 1E2, RTU; Ventana), MUC5AC (clone MRC-19, 1 : 10; Cell Marque, Rocklin, CA, USA), E-cadherin (clone EP700Y, RTU; Cell Marque), and CDX2 (clone EPR2764Y, 1 : 15; Thermo, Rockford, IL, USA).

Histologic sections revealed typical features of hyperplastic gastric polyp, namely, elongated, tortuous, and sometimes cystic gastric foveolae separated by an edematous and inflamed stroma (Figure 1(b)). In addition, the lamina propria was focally infiltrated by groups of atypical epithelial cells, some of them displaying a signet ring appearance (Figure 1(c)). These small aggregates, involving about 10% of the polyp's volume, were consistent with adenocarcinoma. No dysplasia, intestinal metaplasia, or* Helicobacter pylori* was present in the benign gastric epithelium of the polyp. Immunohistochemically, the carcinoma cells reacted strongly and diffusely with cytokeratin 7, estrogen and progesterone receptors, and GATA3 (Figures 1(d) and 1(e)). In contrast, they were negative for cytokeratin 20, CDX2, MUC5AC, and Hep Par 1. E-cadherin displayed membranous staining only in a fraction of the malignant cells. These results supported the diagnosis of a mixed, ductal and lobular carcinoma metastasizing in a gastric hyperplastic polyp.

## 4. Discussion

The occurrence of hyperplastic gastric polyp harboring metastatic carcinoma has not been reported yet. This case involved the extremely rare association of a gastric hyperplastic polyp and focal metastatic breast carcinoma. Histologically, the case could have been diagnosed as primary gastric carcinoma arising in a hyperplastic polyp. However, as the patient had had mammary carcinoma, immunohistochemical stains to analyze the nature of the malignant cell were performed. While markers usually positive in gastric adenocarcinoma such as cytokeratin 20, CDX2, MUC5AC, and Hep Par 1 were negative, those supporting breast carcinoma (estrogen and progesterone receptors, cytokeratin 7, and GATA3) decorated the cancer cells. Accordingly, the case was diagnosed as metastatic breast carcinoma in a gastric hyperplastic polyp. As no other gastric biopsies were taken, there is no certainty regarding the involvement of the nonpolypoid gastric mucosa by metastatic disease. It can be hypothesized, however, that the ascites was rather a result of lobular cancer metastatic to the peritoneum than of a direct overgrowth from the stomach.

Hyperplastic polyps are the most common type of nonneoplastic gastric polyps [9]. Their pathogenesis has not been established but it has been suggested that they may represent a reparative and/or regenerative response to gastric mucosal injury [10]. Histologically they are characterized by hyperplastic, elongated, or dilated foveolar glands within an inflamed and edematous lamina propria [11]. Hyperplastic polyps have been reported in association with various types of chronic gastritis, particularly autoimmune gastritis [11], and* Helicobacter pylori *gastritis [12]. Although they are regarded as benign lesions, development of primary adenocarcinoma may rarely occur, with an incidence ranging between 1.3 and 2.1% [5–8]. Neoplastic transformation of gastric hyperplastic polyps correlates with their size. Han et al. [13] found this process in 12 of 143 polyps >1 cm (8.4%) and in only 2 of 126 polyps <1 cm (1.6). Accordingly, they suggested considering endoscopic polypectomy in hyperplastic polyps >1 cm to achieve an accurate diagnosis. It should be noted that 7 of the polyps with neoplastic transformation were larger than 2.0 cm in diameter.

Metastatic cancer to the stomach is rare. Although virtually all primary neoplasms can metastasize to the stomach, large series of autopsies indicate that in most cases gastric metastases originate from malignant melanomas or carcinomas of the breast, lungs, pancreas, and esophagus [2]. The same sites of origin are most commonly seen in patients who present with gastric metastasis in the clinical setting [4, 14, 15]. Interestingly, two-thirds of mammary carcinoma metastatic to the stomach are of the lobular type [16–18]. Metastatic breast to the stomach leads most frequently to a diffuse mural infiltration (linitis plastica); less frequently, local infiltration in the form of nodules or ulcers can be seen [16]. In fact, at times it may be difficult to endoscopically differentiate between gastric cancer and metastatic breast carcinoma. Moreover, endoscopic biopsies taken from metastatic lobular carcinoma can lead to a misdiagnosis of primary gastric carcinoma as lobular carcinomas may contain large numbers of signet ring cells which otherwise are typically encountered in gastric carcinoma. Avoiding this misdiagnosis is of high importance to establish accurate medical therapy and to prevent an unneeded surgical procedure.

Owing to the morphologic similarity of primary gastric adenocarcinoma and metastatic breast carcinoma on hematoxylin and eosin stained sections, a variety of immunologic markers can be applied in suspicious cases to make this important distinction. Although estrogen and progesterone receptors are typically expressed in breast cancer, about 20% of the cases can be negative [19] and a minority of gastrointestinal carcinomas can be faintly positive [20, 21]. Thus, immunohistochemical analysis for hormonal receptors only is insufficient to prove a diagnosis of metastatic breast carcinoma.

The combination of cytokeratin 7 and cytokeratin 20 has been widely employed to distinguish among different types of carcinoma and it may be useful in distinguishing mammary from gastric carcinoma. While cytokeratin 7 is diffusely and strongly positive in breast cancer, in most gastric carcinomas its reaction is focal and heterogeneous. In contrast, cytokeratin 20 is usually negative in breast carcinoma while gastric cancer cells display a focal and heterogeneous reaction [22]. Our results with both markers (positivity for cytokeratin 7 and negativity for cytokeratin 20) strongly supported a mammary source for the malignant cells.

We added to our immunohistochemical analysis the novel marker GATA3-binding protein, commonly abbreviated as GATA3. This marker stained the cancer cells nicely, with a lack of reactivity in the surrounding benign gastric cells. GATA3, a transcription factor belonging to the GATA family, proved to be a useful immunohistochemical marker for several malignancies, mainly breast and urothelial carcinomas [23, 24]. Miettinen et al. [24], found that 92% and 96% of primary and metastatic mammary ductal carcinomas, respectively, and 100% of mammary lobular carcinoma were diffusely positive for this marker. However, other tumors such as basal cell carcinoma, mesothelioma, and chromophobe carcinoma of the kidney expressed GATA3 as well [23]. Accordingly, as specific markers for breast carcinoma are not available, it is advisable to employ a panel of immunostains to confirm the mammary origin of a metastatic tumor. In our case, all four antibodies supporting breast carcinoma, namely, estrogen and progesterone receptors, GATA 3, and cytokeratin 7, strongly reacted with the cancer cells while the benign gastric cells were negative for all of them.

In summary, to our knowledge this is the first report of carcinoma metastasizing to a hyperplastic gastric polyp. It emphasizes the importance of obtaining a detailed patient history and performing immunohistochemical stains in relevant cases to prevent misdiagnosis and an unnecessary surgical procedure.

## References

[1] Campoli P. M. D. O., Ejima F. H., Cardoso D. M. M., Silva O. Q. D., Santana Filho J. B., Queiroz Barreto P. A. D., Machado M. M., Mota E. D., Araujo Filho J. A., Alencar R. D. C. G., Mota O. M. D. (2006). Metastatic cancer to the stomach. *Gastric Cancer*.

[2] Green L. K. (1990). Hematogenous metastases to the stomach. A review of 67 cases. *Cancer*.

[3] Taal B. G., Peterse H., Boot H. (2000). Clinical presentation, endoscopic features, and treatment of gastric metastases from breast carcinoma. *Cancer*.

[4] de Palma G. D., Masone S., Rega M., Simeoli I., Donisi M., Addeo P., Iannone L., Pilone V., Persico G. (2006). Metastatic tumors to the stomach: clinical and endoscopic features. *World Journal of Gastroenterology*.

[5] Bosseckert H., Kratzsch K. H., Machnik G., Raabe G. (1990). Hyperplasiogenic polyps and stomach carcinoma risk—experiences following 1074 polypectomies and follow-up studies. *Schweizerische Rundschau für Medizin Praxis*.

[6] Daibo M., Itabashi M., Hirota T. (1987). Malignant transformation of gastric hyperplastic polyps. *American Journal of Gastroenterology*.

[7] Hizawa K., Fuchigami T., Iida M. (1995). Possible neoplastic transformation within gastric hyperplastic polyp. Application of endoscopic polypectomy. *Surgical Endoscopy*.

[8] Orlowska J., Jarosz D., Pachlewski J., Butruk E. (1995). Malignant transformation of benign epithelial gastric polyps. *American Journal of Gastroenterology*.

[9] Morais D. J., Yamanaka A., Zeitune J. M. R., Andreollo N. A. (2007). Gastric polyps: a retrospective analysis of 26,000 digestive endoscopies. *Arquivos de Gastroenterologia*.

[10] Jain R., Chetty R. (2009). Gastric hyperplastic polyps: a review. *Digestive Diseases and Sciences*.

[11] Snover D. C. (1985). Benign epithelial polyps of the stomach. *Pathology Annual*.

[12] Ohkusa T., Takashimizu I., Fujiki K., Suzuki S., Shimoi K., Horiuchi T., Sakurazawa T., Ariake K., Ishii K., Kumagai J., Tanizawa T. (1998). Disappearance of hyperplastic polyps in the stomach after eradication of Helicobacter pylori. A randomized, controlled trial. *Annals of Internal Medicine*.

[13] Han A.-R., Sung C. O., Kim K. M., Park C.-K., Min B.-H., Lee J. H., Kim J. Y., Chang D. K., Kim Y.-H., Rhee P.-L., Rhee J. C., Kim J. J. (2009). The clinicopathological features of gastric hyperplastic polyps with neoplastic transformations: a suggestion of indication for endoscopic polypectomy. *Gut and Liver*.

[14] Oda I., Kondo H., Yamao T., Saito D., Ono H., Gotoda T., Yamaguchi H., Yoshida S., Shimoda T. (2001). Metastatic tumors to the stomach: analysis of 54 patients diagnosed at endoscopy and 347 autopsy cases. *Endoscopy*.

[15] Washington K., McDonagh D. (1995). Secondary tumors of the gastrointestinal tract: surgical pathologic findings and comparison with autopsy survey. *Modern Pathology*.

[16] Taal B., Peterse H., Boot H. (2000). Clinical presentation, endoscopic features, and treatment of gastric metastases from breast carcinoma. *Cancer*.

[17] McLemore E. C., Pockaj B. A., Reynolds C. (2005). Breast cancer: presentation and intervention in women with gastrointestinal metastasis and carcinomatosis. *Annals of Surgical Oncology*.

[18] Lamovec J., Bracko M. (1991). Metastatic pattern of infiltrating lobular carcinoma of the breast: an autopsy study. *Journal of Surgical Oncology*.

[19] Chu P. G., Weiss L. M. (2004). Immunohistochemical characterization of signet-ring cell carcinomas of the stomach, breast, and colon. *American Journal of Clinical Pathology*.

[20] Cameron B. L., Butler J. A., Rutgers J., Vargas H. I., Purtell M., Sheppard B. (1992). Immunohistochemical determination of the estrogen receptor content of gastrointestinal adenocarcinomas. *The American Surgeon*.

[21] Yokozaki H., Takekura N., Takanashi A., Tabuchi J., Haruta R., Tahara E. (1988). Estrogen receptors in gastric adenocarcinoma: a retrospective immunohistochemical analysis. *Virchows Archiv—A Pathological Anatomy and Histopathology*.

[22] Chu P. G., Chung L., Weiss L. M., Lau S. K. (2011). Determining the site of origin of mucinous adenocarcinoma: an immunohistochemical study of 175 cases. *The American Journal of Surgical Pathology*.

[23] Ordóñez N. G. (2013). Value of GATA3 immunostaining in tumor diagnosis: a review. *Advances in Anatomic Pathology*.

[24] Miettinen M., McCue P. A., Sarlomo-Rikala M., Rys J., Czapiewski P., Wazny K., Langfort R., Waloszczyk P., Biernat W., Lasota J., Wang Z. (2014). GATA3: a multispecific but potentially useful marker in surgical pathology: a systematic analysis of 2500 epithelial and nonepithelial tumors. *The American Journal of Surgical Pathology*.

